# Comparison of the Delay in Processing Time and Protective Plastic Cases in Two Phosphor Plate Systems

**DOI:** 10.1100/2012/850764

**Published:** 2012-11-07

**Authors:** Ali Murat Aktan, Mehmet Ertuğrul Çiftçi, Faruk Akgünlü

**Affiliations:** ^1^Department of Oral and Maxillofacial Radiology, Gaziantep University, 27310 Gaziantep, Turkey; ^2^Department of of Oral and Maxillofacial Radiology, Selcuk University, 42250 Konya, Turkey

## Abstract

*Objective*. The purpose of this study was to analyze two phosphor plate systems (PSPs) (Dürr Dental, Digora Optime) according to their scanning delay and protective plastic case performances. *Methods*. Two PSPs using an aluminum step wedge were exposed. These plates were placed in three different protective plastic cases (manufacturers' original cases, black case, and white case) before obtaining the radiographs and were then processed immediately and 1, 5, 10, 30, 60, 120, 240, and 480 min after exposure. Mean gray values (MGVs) of the 3rd, 5th, 7th, and 9th steps of the wedges were compared using ANOVA. *Results*. Statistically significant differences were found between the two PSPs (*P* < 0.001). ANOVA revealed that the MGVs of four steps of the wedges were significantly different from each other for all scan delays (*P* < 0.001). MGVs increased with increasing scanning delay, except the group with Dürr plates in their original cases. Reduction in image quality began 5 min after exposure in the other Dürr plate groups. *Conclusions*. Within the limitations of the current study, it can be concluded that scanning delay causes a reduction in image quality, and using the manufacturer's original protective case will result in better performance of PSPs.

## 1. Introduction

Due to advancements in technology, digital radiography is widely used in dental practice [1]. Several systems have been introduced for intraoral digital radiography, and they can be divided into three groups according to their sensor types [2]: Charge-Coupled Device (CCD), Complementary Metal Oxide Semiconductor (CMOS), and Photostimulable Phosphor Plate (PSP) [2]. These digital image receptors are also broadly classified as direct (CCD, CMOS) and indirect (PSP) [3]. In actuality, the PSP system can be referred to as a semidirect imaging system [4].

PSP image receptor systems are distinguished from other types by special features of the plate—thin structure and flexibility—and by the absence of an electrical cord [5, 6], qualities that make it easier to place the receptor into the mount [5]. Other advantages of PSP systems include being available in exactly the same size as conventional film and having a wider dynamic range, which produces better quality radiographs compared with CCD and CMOS sensors [7, 8]. The major disadvantages of PSPs are remaining stored energy in the plate before and even after scanning, lower quality due to repeated use, and the need for additional time for handling and scanning [5, 9, 10]. Several studies have been conducted to compare the quality of images captured using PSPs with images captured using conventional radiography or CCD device [3, 11–16]. Different types of PSP systems have also been evaluated to determine clinical image quality [13, 16–18]. Studies of clinical and laboratory performance of PSPs have been made in regard to caries detection [13, 16, 19], imaging root canals [19], and identification of endodontic instruments [15, 20]. A few investigators have looked at some remaining doubts regarding PSP systems, such as durability of the plate [21], image quality according to scanning delay [14, 22, 23], use of protective plastic cases [2], and storage conditions [24]. However, to our knowledge, no study has focused on the effects on the quality of images acquired using different PSPs enclosed in various protective plastic cases over a series of scanning delay times.

The aim of this study, therefore, was to determine whether the effects of different combinations of protective cases and varying delays in scanning time on digital images acquired using two PSP systems. The null hypothesis was that no differences in image quality could exist between the systems as a result of various scanning delays and use of different protective cases.

## 2. Materials and Methods

In an in vitro study, an aluminium (Al) step wedge was used that was made of 99.5% pure Al and measured 11 mm in total length and 1 mm incremental steps (total of 11 steps). PSP systems were introduced (Vista Scan Combi, Dürr Dental AG, Bietighiem-Bissingen, Germany and Digora Optime, Soradex, Helsinki, Finland) to acquire the digital images. Each PSP image was captured on a new plate, size 2 (30 mm × 40 mm) and acquired on its own scanner at the scanner's highest resolution (40 lp/mm for Vista Scan, and 12.5 lp/mm for Digora Optime). All scanned images were saved as TIFF format. The resulting images were evaluated on a personal computer running the Microsoft Windows XP operating system and using Adobe Photoshop 9.0 (Adobe Systems Inc, San Jose, CA) for the analysis. The exposure settings were determined to see all steps of the aluminium wedge on the radiograph, and this radiograph is considered as gold standard. The plates were exposed for 0.6 seconds at 60 kVp, 10 mA, focus-to-receptor distance 20 cm, using an X-ray unit (Soradex, Helsinki, Finland) with a total filtration equivalent to 1.5 mm Al. An optical bench was used to standardize geometric protection.

Before each exposure, the plates were cleared of any background effect by means of the strong light source built into the scanner. This procedure removed any residual information that may have remained in the plates and thus brought them back to their original state, leaving no memory of previous exposures. After that, the plates were enveloped in one of three different protective plastic cases (black case supplied by the manufacturer, black case supplied by an industry supplier, and white case supplied by an industry supplier).

**Figure 1:**
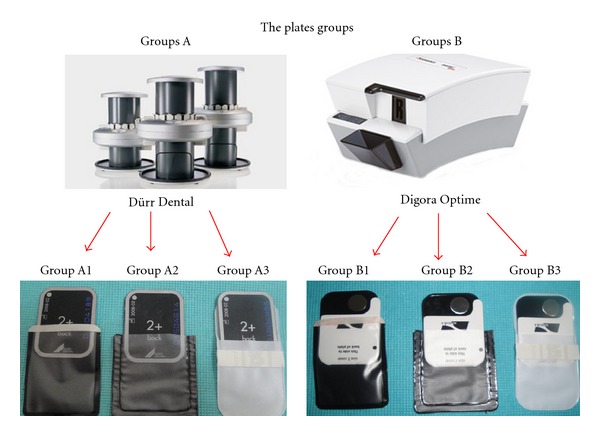
The two main groups (A and B) and three subgroups (1, 2, and 3) for the present study.

The two main groups for this study were the plate groups, one for each scanner. Each plate group had three subgroups, one for each plastic case. In group A, the plates were scanned in the Dürr Dental scanner, and in group B, the plates were scanned in the Digora scanner. Groups A1 and B1 plates were inserted and sealed in the case supplied by the respective manufacturer and kept as such until processing; group A2 and B2 plates were in an industry supplier's black cases; and group A3 and B3 plates were in an industry supplier's white cases (Figure 1). Exposed plates were processed immediately and 1, 5, 10, 30, 60, 120, 240, and 480 minutes after exposure, resulting in nine exposures for each subgroup and a total of 54 exposures for all six subgroups.

**Figure 2:**
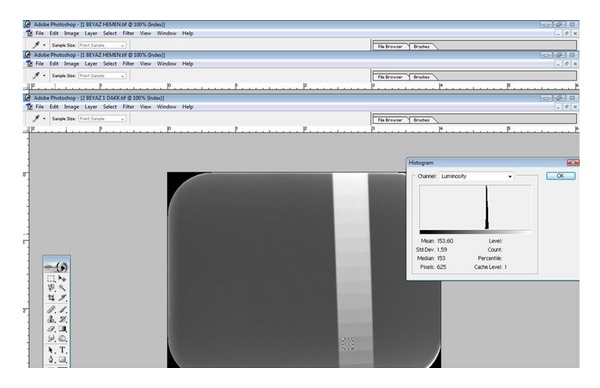
The gray level information of the 3rd step of the Al wedge was sampled with three nonoverlapping (25 × 25 pixel) ROIs using the Photoshop histogram tool.

After all the plates were scanned, the gray level information of the 3rd, 5th, 7th, and 9th steps of the Al wedge was sampled with three nonoverlapping (25 × 25 pixel) regions of interest (ROIs). Black was assigned a value of 0 and white a value of 255 by mean gray values (MGVs), using the Photoshop histogram tool (Figure 2). The MGVs from the immediately scanned plates in each manufacturer's original cases provided the reference standard for each main group.

The data were statistically analysed using the repeated measures analysis of variance (ANOVA) technique (*P* < 0.001). Significance level was set at 5%.

## 3. Results

**Figure 3:**
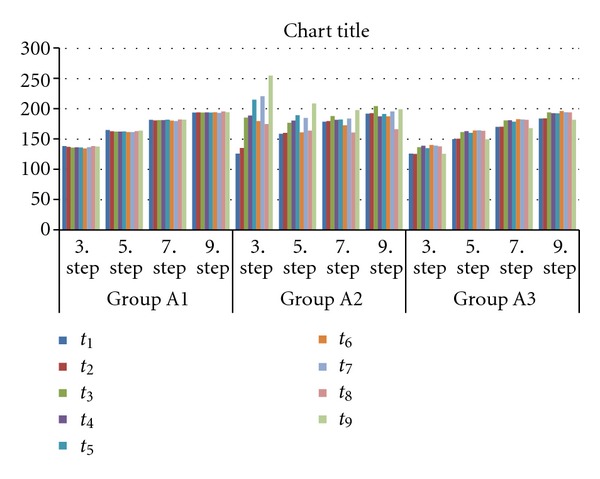
The MGVs of the three subgroups of the Dürr Dental plates according to delay in scanning time.

**Figure 4:**
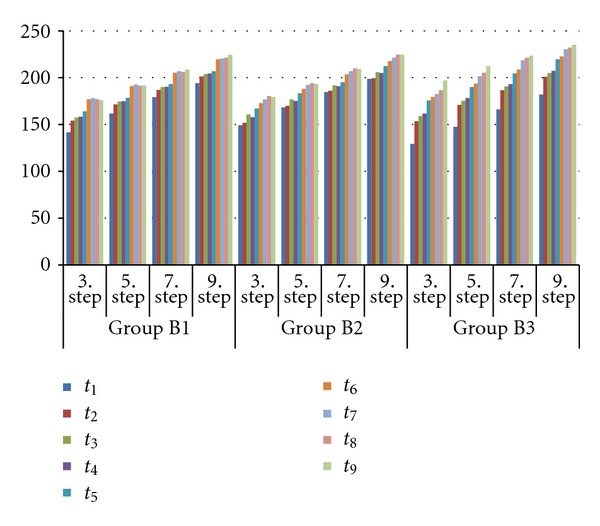
The MGVs of the three subgroups of the Digora Optime plates according to delay in scanning time.

The MGVs of the plates ranged between 125.22 and 254.82 for Group A and 129.38 and 197.12 for Group B. The MGVs of the plates for Groups A and B according to delay in scanning time are presented in Tables 1 and 2, respectively. Multivariate ANOVA revealed interactions between the MGVs of images among times, brands, cases, and steps (Table 3). There were statistically significant differences between the two PSPs (*P* < 0.001). Repeated measures of ANOVA revealed that the MGVs of each step of the Al wedge were significantly different from each other for all scan delays (*P* < 0.001). Figures 3 and 4 depict the tendency for MGVs to increase or decrease according to the delay in scanning time. MGVs increased with increasing scanning delay, except in the group with original Dürr plate cases (Group A1), which showed stable MGV levels with no significant effect of either scanning delay or light source. Reduction in image quality began 5 min after exposure for the other Dürr plate groups (Groups A2 and A3). The subgroups B1, B2, and B3 showed the same tendency when delay in scanning times and type of cases were considered, in contrast to the A subgroups. MGVs from the Digora plates scanned 10 min after exposure were not significantly different from those scanned immediately; however, longer delays showed significant differences in MGVs.

## 4. Discussion

Digital radiography is one of the fastest developing modern dental diagnostic techniques. Serious studies have been conducted to evaluate the diagnostic quality and clinical performance of these systems, PSP being one of the current favourites [1–7, 9, 10, 13–24]. Several investigations have been made into PSP image quality [2, 8, 17, 19, 25], most of them comparisons among PSPs, other digital systems, and conventional radiographic film [3, 11, 26]. Some of the studies only evaluated the clinical and laboratory performance of a single PSP system [21–23, 26], but the literature also has reports comparing the image qualities of different PSP systems [8, 12, 17, 19, 25]. Previous studies have demonstrated that one of the main reasons behind the fading of the latent image in a PSP system is the delay in scanning time. Moreover, previous studies have been conducted on PSP scanning time, but the results have been for one system only. In the present study, two PSP systems were evaluated, comparing the effects of processing delay.

The current study also examined the performance of three different protective plastic cases in each of the two PSP systems. There are few reports on this subject [2, 24]. Martins et al. [24] believed that plates can be processed within 6 hours if stored in the appropriate cases. Bramante et al. [2] showed that plastic cases supplied by the manufacturer provided better protection to the plates than other cases. The result of this study was in agreement with the previous report. According to the results of the current study, Dürr Dental's original case provided adequate protection from the light source, irrespective of the length of delay in scanning time, unlike the other black and white cases. Increases in MGVs for the Digora plates were found when delay in scanning time increased, irrespective of which protective plastic cases were used. Hence, the findings of the present study suggest that the original plastic case should be used to acquire better image quality when using Dürr Dental plates, while any of the three cases tested can be used to acquire reasonable diagnostic quality when using Digora plates. These results allow alternate cases to be used for Digora plates but not for Dürr Dental plates and also suggest that when the plates are exposed to the light source, Dürr Dental plates are more negatively affected than Digora plates.

Some authors who researched the delay in PSP scanning time emphasized that Digora plates should be scanned within 10 min of exposure [23]. Others concluded that the gray level values of the background (as well as those of the steps of the Al wedge) in plates scanned after a half of delay in scanning time were not different from those in immediately scanned plates [22]. Bramante et al. [2] stated that the processing delay of 120 min for Digora plates caused a reduction in image quality. Martins et al. [24] reported that the PSP started to lose information within 5 min of image capture and that almost half of the information was lost within 1 h. The results of the present study revealed that the small differences in MGV for each Dürr Dental plate in its original case are not statistically significant, irrespective of increase of processing delay; however, for Dürr Dental plates in the black or white cases provided by industry suppliers, differences are statistically significant, respective to the length of the processing delay.  Figure 3 shows that Dürr Dental plates in black or white cases provided by industry suppliers had irregular graphics, with information loss starting within 5 min after exposure. The differences in MGVs for Digora plates varied for each of the cases used in this study; reduction in image quality started within 10 min after exposure for each case. 

Another result of the present study was that the gray levels of each step of the Al wedge differed significantly from each other for all scan delays and cases. This result shows that protective cases provide proper protection of the whole plate surface from the effect of light, and protection provided by the original cases was better than that provided by the other black and white cases.

In the present study, plates were neither in a light box nor in a dark drawer, in order to prevent any external light energy from affecting the trapped electrons. Although those procedures were followed in previous studies on the effect of delay in scanning time on PSP image quality [22], they were not followed in the present study because they are not always carried out in clinical practice, and the preferred aim was to achieve accurate simulation to evaluate the effects of delay in scanning time. This study was conducted to see whether delay in scanning and the use of different cases would result in loss of image quality in clinical practice.

In the current study, better image quality was obtained in Dürr Dental plates enclosed in their original cases before exposure. It was demonstrated that original cases perform better in protecting plates from light for up to 8 h and should therefore be preferred when Dürr Dental plates are used. However, when Digora plates were exposed to light, the effect did not depend on the type of case used. In addition, when the plates were exposed to light, a reduction in image quality was seen up to 5 and 10 minutes after exposure for the Dürr Dental and Digora Optime PSPs, respectively. 

## Figures and Tables

**Table 1:** The MGVs of the plates for Group A according to delay in scanning time.

	Group A (Dürr Dental)											
	Group A1				Group A2				Group A3			
	3. step	5. step	7. step	9. step	3. step	5. step	7. step	9. step	3. step	5. step	7. step	9. step
*t*_1_	138,42	164,78	181,76	193,80	125,87	158,55	178,73	192,18	125,87	150,03	169,94	183,96
*t*_2_	137,11	162,84	180,68	194,08	135,28	160,39	179,80	192,56	125,22	150,49	170,28	184,21
*t*_3_	135,64	162,34	181,32	193,55	185,63	176,81	188,26	204,46	136,63	161,68	180,59	193,84
*t*_4_	136,40	162,37	181,24	193,98	188,73	180,31	181,81	187,55	138,91	163,09	181,18	192,66
*t*_5_	136,09	162,44	181,90	193,52	214,97	189,53	182,17	191,24	134,96	160,35	178,85	192,49
*t*_6_	134,42	161,60	180,49	194,15	179,78	161,02	172,76	187,47	140,11	164,15	182,89	196,14
*t*_7_	136,53	161,64	179,78	193,29	220,92	184,93	184,10	195,67	139,28	164,49	182,22	194,42
*t*_8_	138,20	163,16	182,17	195,71	174,98	163,83	160,51	166,16	137,83	163,66	181,70	194,04
*t*_9_	137,65	163,92	182,09	194,38	254,82	208,86	197,96	199,05	125,79	149,58	168,08	181,69

*t*_1_: immediately, *t*_2_: 1 minutes, *t*_3_: 5 minutes, *t*_4_: 10 minutes, *t*_5_: 30 minutes, *t*_6_: 60 minutes, *t*_7_: 120 minutes, *t*_8_: 240 minutes, *t*_9_: 480 minutes.

**Table 2:** The MGVs of the plates for Group B according to delay in scanning time.

	Group B (Digora Optime)											
	Group B1				Group B2				Group B3			
	3. step	5. step	7. step	9. step	3. step	5. step	7. step	9. step	3. step	5. step	7. step	9. step
*t*_1_	141,55	161,66	179,23	194,25	149,19	168,20	184,63	198,91	129,38	147,62	166,17	182,17
*t*_2_	153,99	171,48	187,05	201,36	151,73	169,95	186,11	199,54	153,49	170,94	186,59	200,89
*t*_3_	157,52	174,59	189,86	203,88	160,59	176,87	191,88	206,09	158,94	175,29	190,56	204,98
*t*_4_	158,23	174,70	190,33	204,49	157,89	175,15	190,85	205,04	161,73	178,21	193,07	207,17
*t*_5_	163,93	178,63	193,07	206,80	167,11	183,17	195,32	212,34	175,56	189,94	204,71	219,70
*t*_6_	176,99	191,09	205,34	219,63	172,82	188,00	203,60	217,88	179,35	193,82	208,69	222,62
*t*_7_	178,14	192,66	207,05	220,57	176,77	192,17	206,96	221,44	182,59	201,55	218,69	230,46
*t*_8_	177,07	191,40	206,26	221,36	180,51	194,16	209,87	224,80	186,51	205,41	221,36	232,22
*t*_9_	176,04	191,73	208,82	224,56	179,48	193,33	209,45	224,80	197,13	212,41	223,62	235,02

*t*_1_: immediately, *t*_2_: 1 minutes, *t*_3_: 5 minutes, *t*_4_: 10 minutes, *t*_5_: 30 minutes, *t*_6_: 60 minutes, *t*_7_: 120 minutes, *t*_8_: 240 minutes, *t*_9_: 480 minutes.

**Table 3:** Multivariate test results based on four factor repeated measures ANOVA for changes in MGVs.

Effect		Value	*F*	Hypothesis df	Error df	Sig.
Time	Pillai's Trace	,999	3661,947^a^	8,000	40,000	,000
Time ∗ Brend	Pillai's Trace	,996	1117,755^a^	8,000	40,000	,000
Time ∗ Case	Pillai's Trace	1,977	432,353	16,000	82,000	,000
Time ∗ Step	Pillai's Trace	1,771	7,562	24,000	126,000	,000
Time ∗ Brend ∗ Case	Pillai's Trace	1,955	223,032	16,000	82,000	,000
Time ∗ Brend ∗ Step	Pillai's Trace	1,842	8,350	24,000	126,000	,000
Time ∗ Case ∗ Step	Pillai's Trace	2,719	4,660	48,000	270,000	,000
Time ∗ Brend ∗ Case ∗ Step	Pillai's Trace	2,340	3,596	48,000	270,000	,000

^a^Exact statistic.

## References

[1] Melo DP, Pontual AA, Almeida SM, Campos PF, Tosoni GM (2009). Alternative erasing times of the DenOptix system plate: performance on the detection of proximal caries. *Oral Surgery, Oral Medicine, Oral Pathology, Oral Radiology and Endodontology*.

[2] Bramante CM, Bramante AS, De Souza RE, Moraes IG, Bernardineli N, Garcia RB (2008). Evaluation of the effects of processing delays and protective plastic cases on image quality of a photostimulable phosphor plate system. *Journal of Applied Oral Science*.

[3] Athar A, Angelopoulos C, Katz JO, Williams KB, Spencer P (2008). Radiographic endodontic working length estimation: comparison of three digital image receptors. *Oral Surgery, Oral Medicine, Oral Pathology, Oral Radiology and Endodontology*.

[4] Versteeg CH, Sanderink GCH, Van Der Stelt PF (1997). Efficacy of digital intra-oral radiography in clinical dentistry. *Journal of Dentistry*.

[5] Ergün S, Güneri P, Ilgüy D, Ilgüy M, Boyacioğlu H (2009). How many times can we use a phosphor plate? A preliminary study. *Dentomaxillofacial Radiology*.

[6] Van Der Stelt PF (2005). Filmless imaging: the uses of digital radiography in dental practice. *Journal of the American Dental Association*.

[7] Brennan J (2002). An introduction to digital radiography in dentistry. *Journal of Orthodontics*.

[8] Berkhout WER, Beuger DA, Sanderink GCH, Van Der Stelt PF (2004). The dynamic range of digital radiographic systems: dose reduction or risk of overexposure?. *Dentomaxillofacial Radiology*.

[9] Ang DB, Angelopoulos C, Katz JO (2006). How does signal fade on photo-stimulable storage phosphor imaging plates when scanned with a delay and what is the effect on image quality?. *Oral Surgery, Oral Medicine, Oral Pathology, Oral Radiology and Endodontology*.

[10] Kashima I (1995). Computed radiography with photostimulable phosphor in oral and maxillofacial radiology. *Oral Surgery, Oral Medicine, Oral Pathology, Oral Radiology and*.

[11] Bhaskaran V, Qualtrough AJE, Rushton VE, Worthington HV, Horner K (2005). A laboratory comparison of three imaging systems for image quality and radiation exposure characteristics. *International Endodontic Journal*.

[12] Hintze H, Wenzel A, Frydenberg M (2002). Accuracy of caries detection with four storage phosphor systems and E-speed radiographs. *Dentomaxillofacial Radiology*.

[13] Møystad A, Svanaes DB, Risnes S, Larheim TA, Gröndahl HG (1996). Detection of approximal caries with a storage phosphor system. A comparison of enhanced digital images with dental X-ray film. *Dentomaxillofacial Radiology*.

[14] Oliveira AE, de Almeida SM, Paganini GA, Neto FH, Bóscolo FN (2000). Comparative study of two digital radiographic storage phosphor systems. *Brazilian dental journal*.

[15] Ong EY, Ford TRP (1995). Comparison of radiovisiography with radiographic film in root length determination. *International Endodontic Journal*.

[16] Svanaes DB, Møystad A, Risnes S, Larheim TA, Gröndahl HG (1996). Intraoral storage phosphor radiography for approximal caries detection and effect of image magnification comparison with conventional radiography. *Oral Surgery, Oral Medicine, Oral Pathology, Oral Radiology, and Endodontics*.

[17] Kitagawa H, Farman AG, Scheetz JP (2000). Comparison of three intra-oral storage phosphor systems using subjective image quality. *Dentomaxillofacial Radiology*.

[18] Borg E, Gröndahl HG (1996). On the dynamic range of different X-ray photon detectors in intra-oral radiography. A comparison of image quality in film, charge-coupled device and storage phosphor systems. *Dentomaxillofacial Radiology*.

[19] Shearer AC, Mullane E, Macfarlane TV, Gröndahl HG, Horner K (2001). Three phosphor plate systems and film compared for imaging root canals. *International Endodontic Journal*.

[20] Shearer AC, Horner K, Wilson NH (1991). Radiovisiography for length estimation in root canal treatment: an in-vitro comparison with conventional radiography. *International Endodontic Journal*.

[21] Bedard A, Davis TD, Angelopoulos C (2004). Storage phosphor plates: how durable are they as a digital dental radiographic system?. *Journal of Contemporary Dental Practice*.

[22] Akdeniz BG, Gröndahl HG (2006). Degradation of storage phosphor images due to scanning delay. *Dentomaxillofacial Radiology*.

[23] Akdeniz BG, Gröndahl HG, Kose T (2005). Effect of delayed scanning of storage phosphor plates. *Oral Surgery, Oral Medicine, Oral Pathology, Oral Radiology and Endodontology*.

[24] Martins MGBQ, Neto FH, Whaites EJ (2003). Analysis of digital images acquired using different phosphor storage plates (PSPs) subjected to varying reading times and storage conditions. *Dentomaxillofacial Radiology*.

[25] Borg E, Attaelmanan A, Gröndahl HG (2000). Image plate systems differ in physical performance. *Oral surgery, Oral Medicine, oral Pathology, Oral Radiology, and Endodontics*.

[26] Cederberg RA, Frederiksen NL, Benson BW, Shulman JD (1998). Effect of different background lighting conditions on diagnostic performance of digital and film images. *Dentomaxillofacial Radiology*.

